# Helminth–HIV Coinfection: Should We Deworm?

**DOI:** 10.1371/journal.pntd.0000160

**Published:** 2007-12-19

**Authors:** Gadi Borkow, Carrie Teicher, Zvi Bentwich

**Affiliations:** 1 Cupron, Beth-Shemesh, Israel; 2 Center for Infectious Tropical Diseases and AIDS, Faculty of Health Sciences, Ben Gurion University, Beer Sheba, Israel; London School of Hygiene & Tropical Medicine, United Kingdom

The prevalence of helminthic infections in most of the developing countries is overwhelming, and almost a quarter of the world's population is infected by them. Likewise, with the spread of the AIDS epidemic, dual helminthic and HIV-1 coinfections are extremely common, particularly in Africa [1]. Several years ago we suggested that helminth infections play a major role in the pathogenesis of HIV-1 infection in Africa and other developing countries, due to their profound effects on the host immune system, which would make those infected with helminths more susceptible to HIV-1 infection and more vulnerable to the disease's effects [2]. As summarized in three more recent reviews, we and others have demonstrated that chronic immune activation with a dominant T helper cell 2 profile, and anergy, are indeed the hallmarks of chronic helminth infection [3]–[5]. These immune changes are characterized by several modulations in the normal immune response, particularly those of the cellular immune response, which together could account for possible profound effects of the chronic helminthic infection on the host's ability to handle HIV as well as other infections. Importantly, most of these modulations are reverted almost completely to normal following treatment of the helminthic infections.

Several studies have since lent further support to this association: (i) T cells and monocytes obtained from helminth-infected individuals have increased expression of HIV-1 chemokine coreceptors [6]–[8]; (ii) peripheral blood mononuclear cells obtained from helminth-infected individuals are more susceptible to HIV-1 infection, and the increased susceptibility is correlated to the chronic immune activation of the cells [9],[10]; (iii) the risk of mother-to-child HIV-1 transmission in pregnant women coinfected with one or more helminths was found to be significantly higher than in women without helminth infections [11]; (iv) an association was found between genital lesions caused by *Schistosoma haematobium* infection in women and susceptibility to HIV infection [12]; and (v) people coinfected with HIV and *S. mansoni* have decreased CD8^+^ cytolytic HIV-1-specific T cell responses and increased interleukin-10 production compared to individuals infected with HIV-1 only [13].

Perhaps more compelling with regard to the effect helminth infection may have on HIV infection are the results of recent studies in primates dually infected with *Schistosoma* and simian-human immunodeficiency virus (SHIV), which show that: (i) *Schistosoma*-infected monkeys required significantly less SHIV to become infected with the virus in comparison to *Schistosoma*-noninfected animals [14]; (ii) primates infected with *S. mansoni*, when acutely infected with SHIV, developed higher levels of plasma SHIV in comparison with *Schistosoma*-noninfected animals, and regardless of the intensity of the parasite infection; (iii ) re-exposure to *Schistosoma* of monkeys infected with SHIV resulted in additional significant increase of the SHIV viral load [14],[15]; and (iv) primates chronically infected with SHIV, when also infected with *Schistosoma,* showed a significant increase of SHIV plasma viral load together with a decrease in the percentage of their CD4(^+^) CD29(^+^) memory cells [16].

With this background, Walson and John-Stewart have performed a systematic review of the literature on studies determining the effects of treatment of helminth coinfection in HIV-1-infected individuals, which is published in this issue [17]. They identified 6,384 relevant citations of which only 15 were identified as potentially relevant studies; at last only five studies were found eligible for inclusion based on the HIV/AIDS Cochrane Review Group search strategy and guidelines. Most studies were not included due to inadequate reporting or collection of data, lack of a control comparison group, and failure to confirm helminth infection status. Among the five studies finally included in the analysis, only one is a single randomized controlled trial and the other four are observational studies. As a result of their analysis, Walson and John-Stewart conclude that despite the void in conclusive evidence demonstrating the efficacy of anti-helminth treatment in slowing HIV-1 disease progression, there is a positive outcome in regard to the correlation between helminth infection treatment and HIV plasma viral load decrease (though no correlation was observed with other relevant parameters, such as CD4 T cell counts) in the five very carefully reviewed studies. The fact that only five studies, out of over 6,000 reviewed studies addressing this issue, were finally included in the analysis clearly illustrates the paucity of good studies in this area. Furthermore, even within this small group of studies, significant differences in methodology and evaluation are evident, and therefore conflicting results are not surprising (let alone the lack of significant effects of deworming on CD4 levels). Thus, Walson and John-Stewart rightly conclude from their review that there is an urgent need for larger and wider studies of this question, and that they should be preferably double-blinded and well controlled. It is of interest to note that since the time the review has been performed there has been one additional report of a study performed in Tanzania, which showed significant effects of eradication of filarial infection on plasma HIV viral load decrease [18].

While overall we fully agree with these conclusions, we wish to re-emphasize two major issues. First, given that the immune changes accompanying helminth infections are profound and universal, and more importantly, that they revert to normal after eradication of the helminth infection, their role in the interaction between the host and the other infections cannot be overemphasized and should therefore be adequately addressed in any future design of studies on treatment of helminths in dual infection with HIV. Second, helminth infections have an ongoing prolonged and wide negative effect on the health of the population, irrespective of their effects on HIV and possibly other infections as well. Thus, control of helminths should be pursued in large parts of the developing world without delay and should not await the results of studies on the impact of helminths on HIV or other infections. Moreover, since control of all neglected tropical diseases has become a feasible and attainable objective, and is now offered as a “package” treatment [19], its wide application can even take precedence over studies on the specific effects of deworming on HIV viral load. Taking this broad approach may still help determine the effects deworming will have on the epidemiology and spread of HIV and other infections, as well as shedding further light on the role of host immunity in this interaction and on susceptibility to HIV infection. Lastly, such an approach does not have to exclude any well-designed and large-scale studies that will address specific issues referred to above, as Walson and John-Stewart recommend.
